# The gut is guilty! Will legalomics transform forensic and legal psychology?

**DOI:** 10.3389/fpsyg.2026.1739593

**Published:** 2026-03-18

**Authors:** Pragya Mishra, Susan L. Prescott, Alan C. Logan

**Affiliations:** 1 Department of Law, University of Allahabad, Prayagraj, Uttar Pradesh, India; 2 School of Medicine, University of Western Australia, Perth, WA, Australia; 3 Department of Family and Community Medicine, University of Maryland School of Medicine, Baltimore, MD, United States; 4 Center for Justice and Mental Wellbeing, Nova Institute for Health, Baltimore, MD, United States

**Keywords:** auto-brewery syndrome, forensic psychology, gut–brain axis, legalomics, Mendelian randomization, microbiome, neurorights, psychobiotics

## Abstract

Multiple lines of converging research are supporting the idea that gut microbes play an outsized role in human cognition and behavior. Here in this perspective article, we argue that emergent gut-brain-microbiota research, and associated advances in multi-omics technologies, are destined to be of high-level relevance to forensic and legal psychology. After summarizing neural, immune, endocrine, and metabolic channels by which gut ecosystems can modulate behavior-relevant brain states, and discussing causal inferences from microbiota-transfer and adjacent human evidence, we present auto-brewery syndrome as a bounded legal precedent for microbiome-mediated impairment. The available evidence allows for a visualized future in which legalomics—the disciplined use of microbiome and omics evidence in prevention, treatment, competency, mitigation, risk assessments, reintegration care, correctional health, and professional wellness—is in the prevue of forensic and legal psychology. Framed by neurorights, we offer a series of ideas for future directions, with possible ways to strengthen research within ethical frameworks. Using auto-brewery syndrome as an example, we argue that the legalome offers forensic and legal psychology a way to calibrate, rather than replace, biopsychosocial judgement. Microbial signatures and legalomics—reliably obtained and narrowly construed—might one day help us judge more justly.

## 1 Introduction

In the Global North, forensic psychology and psychiatry largely operate within Anglo-American criminal-law frameworks organized around desert-based punishment, retribution, and folk-psychological assumptions about free will, stable character, and willpower. Outside narrow exceptions (e.g., legal insanity or severe neurodevelopmental disability), the idealized legal agent is presumed a rational chooser whose conduct reflects personal traits and intentions. Yet emergent evidence challenges these prescientific assumptions, illuminating the extent to which biology can constrain agency (Callender, 2021; Sapolsky, 2024; Logan and Prescott, 2025). The burgeoning field of neurolaw—and its neurorights discourse—forces this tension into view (Logan and Mishra, 2025).

Converging evidence across neuroscience and psychobiology shows that cognition, affect, impulse control, interoception, and stress reactivity are influenced by trillions of microorganisms inhabiting the human gastrointestinal tract (Hashimoto, 2023). Under defined conditions, these “external yet intimate” influences have the potential to shift behavior-relevant brain states in ways that matter for the legal system, prompting consideration of their place in culpability, mitigation, competency, correctional practice, prevention of justice involvement, and professional wellness (Mishra et al., 2025).

In this Perspective, we summarize gut–brain mechanisms; highlight causal leverage from microbiota-transfer and human intervention studies; consider auto-brewery syndrome as a bounded precedent; and outline practice applications with neurorights-aware guardrails. We introduce the emerging concept of the legalome, defined as the application of microbiome sciences and objective omics-derived markers in forensic and legal psychology. The legalome describes the biological ecology of justice. It is distinct from the type of forensic microbiology that could, for example, aid in human identification, place a perpetrator at a crime scene, establish postmortem intervals, and/or help establish a cause of death. Instead, the legalome focuses on the potential explanatory power of microbes and microbe-derived metabolites (and intersection with other ‘omics' markers - e.g., genomics, metabolomics, epigenomics, and transcriptomics) in the context of cognition and behaviors that might otherwise lead to, or be associated with, justice involvement (Logan et al., 2025a). The legalome brings objectivity to what might be described as forensic or legal neuroecology. That is, it includes consideration of the ways in which the human microbiome acts as a biological transducer of multiple environmental inputs, ultimately intersecting with brain architecture and function.

As noted above, the legalome includes applications outside of forensic psychology. It is also situated within the broader realms of legal psychology. This latter field is concerned with all actors within systems of criminal justice—encompassing the behavior, lifestyle and fitness-for-duty of law enforcement personnel, judges, lawyers, corrections, and forensic professionals. All of these actors are engaged in high-stakes, high-stress, working environments wherein occupational burnout rates are high (Costanzo and Krauss, 2021). The consequences of burnout in criminal justice work extend far beyond the individual professional (Queirós et al., 2013; Chlap and Murray, In press). The integration of multi-omics and microbiome markers into legal psychology has the potential to bring objectivity to a realm that has been reliant upon ‘paper-and-pencil' self-report and the subjective impressions of clinicians (Oprinca-Muja et al., 2026).

**Figure 1:**
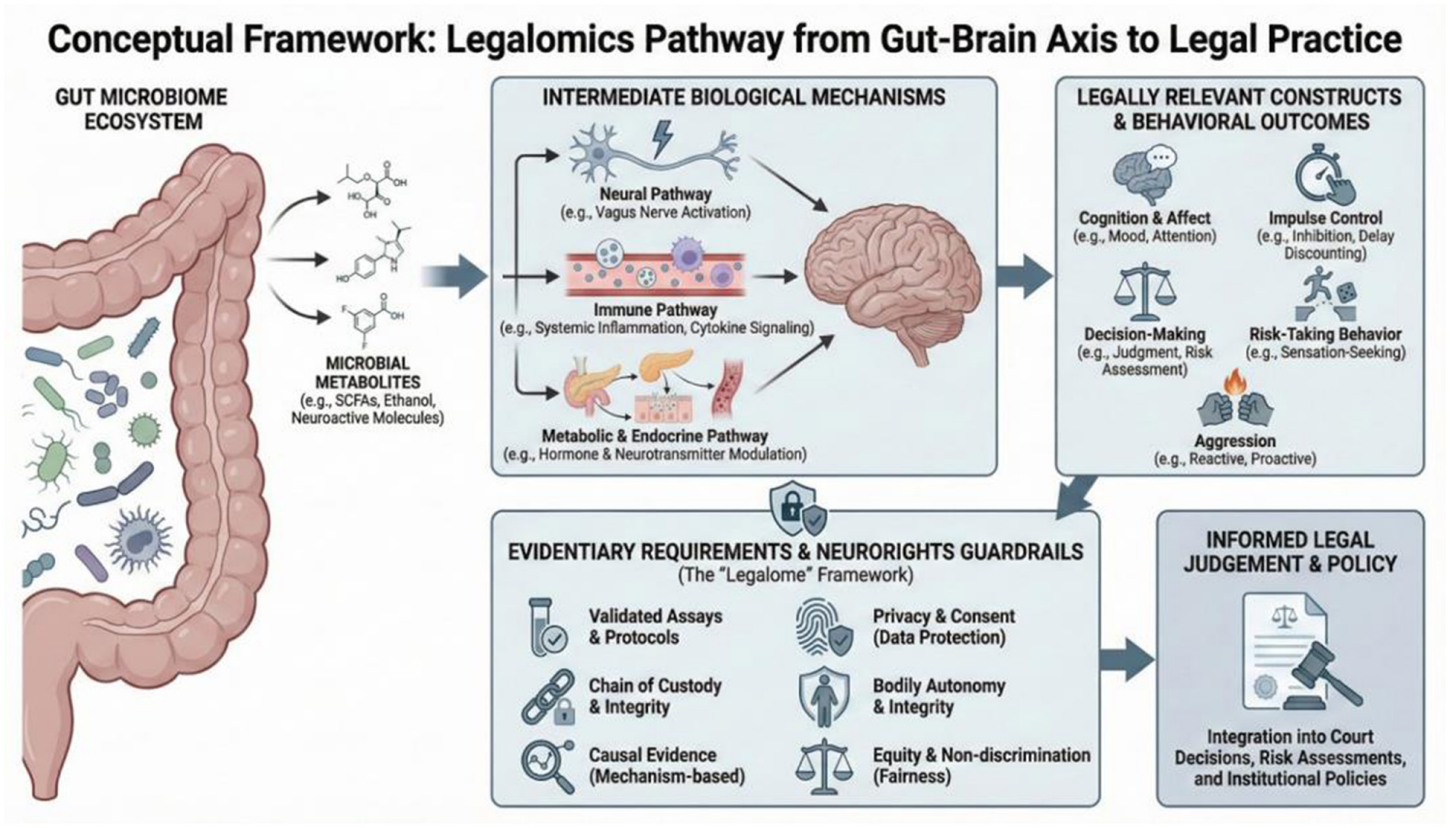
The legalome framework: as mechanistic, preclinical and clinical research continues to point toward microbiota as contributors to cognition and behavior otherwise associated with justice involvement, it is critical to consider the possible legal and ethical implications. Image credit: David H. Nelson, used with permission.

At the outset, we underscore that the emergent research remains at the nascent stage. In this perspective our principal argument is that if the disparate evidence described below continues to mature and coalesce, it will help inform court decisions, risk and wellness assessments, and institutional policies. It is understood that the field is dominated by preclinical work. There is a need for translation science and causal evidence that provides confidence that microbes are a unique explanatory marker of various states of human wellbeing (Metwaly et al., 2025). At the same time, though, there is enough existing evidence to take seriously the need for scientists and professionals in the fields of criminal justice to be engaged in shaping future research, and evaluating the positive benefits and ethical conundrums that may arise. As we describe below, the microbe-driven condition of auto-brewery syndrome is already presenting challenges to courtroom assumptions of intent and culpability. Our position here does not assume biological determinism; the aim is to locate proportionate, evidence-aligned uses where credible biology can calibrate (not replace) judgement to improve fairness, accuracy, care, and professional wellness within justice systems (Figure 1).

## 2 Gut microbiome, brain, and the self

The human gut microbiome comprises trillions of gastrointestinal microorganisms and the genes and metabolites through which they interact with the host. Once a fringe proposal (Logan and Katzman, 2005; Logan et al., 2003), convergent evidence now maps neural, immune, metabolic, and endocrine routes by which gut ecosystems influence brain structure and function (Devason et al., 2024; Doenyas et al., 2025).

### 2.1 Neural signaling

The vagus provides a conduit from gut to limbic circuits; in animal models, behavioral effects of select live biotherapeutics disappear after vagotomy, indicating a causal vagal route into stress- and affect-related circuits (Cryan and Dinan, 2012).

### 2.2 Metabolites and endocrine crosstalk

Microbes shape nutrient absorption and generate or transform neuroactive molecules—SCFAs, tryptophan metabolites, and bile-acid derivatives—that reach the brain and modulate neurotransmission, plasticity, and epigenetic programming (Hull and Sun, 2025; Taherkhani et al., 2025). Microbial control of bile acids is established (Sayin et al., 2013). SCFAs activate FFAR2/FFAR3 (GPR43/GPR41) on enteroendocrine cells, augmenting GLP-1 and PYY with downstream neuroendocrine and behavioral effects (Zhou et al., 2023; Grundeken and El Aidy, 2025).

### 2.3 Immune and barrier pathways

Microbiome states maintain mucosal integrity; perturbation increases intestinal permeability, allowing products such as lipopolysaccharide endotoxin (LPS) to drive low-grade systemic inflammation (measured via CRP, IL-6, TNF-α), neuroinflammatory signaling, corticolimbic changes, and HPA-axis effects on cognition, mood, aggression, and decision-making (Morena et al., 2025; Warren et al., 2024; Chen et al., 2025; Castle et al., 2021; Yu Y. et al., 2024).

Taken together, these mechanisms situate agency within organism–environment dynamics—including microbial ecologies—and support accounts of gut microbes as “transducers” of environmental forces that challenge traditional concepts of self and selfhood (Rees et al., 2018a; Kundu et al., 2017; Rees et al., 2018b).

## 3 Microbiota-transfer studies and causation

A direct way to probe causality is to transfer a screened donor's gut microbial community into a recipient whose intestinal ecosystem is made receptive (germ-free or antibiotic-conditioned). The conditions of either the donor or recipient can be manipulated (e.g., high-fat diet, environmental toxin exposure). As a research tool this is termed fecal microbiota transplantation (FMT) or microbiota transfer. In neuropsychiatry contexts, recipients are then observed for behavioral and physiological change.

### 3.1 Preclinical FMT

Across international laboratories, donor communities shift recipient behavior. Classic exchanges between timid BALB/c and bold NIH Swiss mice led recipients to adopt donor-like exploratory–anxious profiles (Bercik et al., 2011). Microbiota from animals exposed to chronic alcohol increase depression-, anxiety-, and aggression-like behaviors in recipients (Shen et al., 2024), while microbiota from human infants exposed to antibiotics increases aggression in recipient animals (Uzan-Yulzari et al., 2024). Conversely, transfer from resilient or “healthy” donors attenuates drug-seeking and withdrawal, reduces stress-related behaviors, and improves cognition after early-life adversity (Saeedi et al., 2025; Dong et al., 2024). Humanized-mouse designs extend the pattern: material from adults with mood disturbance, cognitive impairment, or substance-use disorders significantly influences recipient behaviors (Xiao et al., 2021; Wei et al., 2024; Kelly et al., 2016; Wolstenholme et al., 2022; Wang T. et al., 2024; Cai et al., 2025).

### 3.2 Biological correlates

Behavioral shifts co-occur with altered CNS gene expression and neurotrophic signaling (e.g., BDNF), changes in serotonergic and GABAergic pathways, and immune–metabolic signatures of heightened inflammatory tone (Logan et al., 2025b; Chang et al., 2024). Transfers using human donors with postpartum depression show elevated circulating inflammatory signals and central neuroinflammatory read-outs alongside depressive-type behaviors (Cao et al., 2025).

### 3.3 Human translation

Early human FMT studies report benefits in mood, alcohol-use disorder (craving/consumption), autism-related gastrointestinal and behavioral domains, and sleep (Borrego Ruiz, 2024; Fang et al., 2023; Hazan et al., 2024; Bajaj et al., 2021; He et al., 2024), along with metabolic improvements (e.g., insulin sensitivity, lipid profiles) in defined cohorts, including multi-year durability reports in obesity research (Wilson et al., 2025). Protocols vary (donor selection, preparation, dosing). The path forward is preregistered, adequately powered RCTs with standardized collection, chain-of-custody, and laboratory quality systems to calibrate effect sizes and specify which profiles benefit—and when.

## 4 Additional causal support

Beyond microbiota-transfer studies, human-facing evidence adds causal leverage. Two lines dominate: targeted interventions (probiotics, prebiotics, diet) and genetic causal inference via Mendelian randomization (MR).

### 4.1 Human interventions

Meta-analyses of randomized trials show modest but meaningful improvements in mental health and cognition with select preparations (Ma H. et al., 2025; Zandifar et al., 2025). Early studies also report improvements on aggression-related outcomes (aggressive behavior, thoughts, irritability/anger) (Montazeri et al., 2025; Eastwood et al., 2025; Steenbergen et al., 2015; Huang et al., 2025; Marotta et al., 2019), risk-taking/impulsivity (Roman et al., 2018; Rojo-Marticella et al., 2025; Ruiz-Gonzalez et al., 2024; Dantas et al., 2022; Falkenstein et al., 2024), and emotion regulation/facial affect recognition (Niu et al., 2025; Baião et al., 2023).

### 4.2 Mendelian randomization

Recent MR analyses implicate gut-microbial taxa and functions in depression, schizophrenia, bipolar spectrum, and addiction-related phenotypes, with inflammatory mediators (e.g., CRP/IL-6 signaling) as plausible biological bridges (Fatoba and Simpson, 2025; Zeng et al., 2024; Zhao et al., 2025; Wang X. et al., 2024). Signals are bidirectional in places (psychiatric liability → microbial composition), underscoring a dynamic host–microbe system. While instrument strength and horizontal pleiotropy remain technical considerations, the MR corpus strengthens the case that gut-linked pathways are causally relevant to behavior-salient brain states (Ma Z. et al., 2025).

## 5 Auto-brewery syndrome: legal precedent for microbiome-mediated impairment

One of the most obvious examples of forensic neuroecology and the legalome in action is the condition known as auto-brewery syndrome (ABS), a condition in which gut microbiota (through fermentive action on dietary carbohydrates) manufacture significant quantities of ethanol. Here, the individual can unknowingly experience elevated blood and breath alcohol levels in ranges associated with cognitive impairment, impulsivity, lowered inhibition, and/or irritability. At the extreme, the condition can lead to systemic elevations in blood ethanol that are far beyond legal standards for driving while intoxicated (DUI). While once thought to be an ultra-rare condition with little forensic relevance, emerging evidence demonstrates that the underlying mechanisms that create the dysbiotic conditions for ABS (i.e., overgrowth of ethanol producing microbes such as *Candida spp*., *Klebsiella pneumoniae*, and *Escherichia coli*) are not rare (Hsu et al., 2026; Hsu and Schnabl, 2026; Singh et al., 2025).

Courts in the United States, Belgium, and the Netherlands, have already adjudicated several ABS-related DUI cases (Logan et al., 2026). In these rare cases, judges have recognized protocol-driven evidence consistent with ABS—typically supervised carbohydrate challenges showing reproducible post-prandial rises in blood or breath alcohol—leading to dismissals or charge reductions, narrowed culpability, or findings of involuntary intoxication. Some of these cases have received international media attention and reporting by trusted news outlets (Herbeck, 2016; Nelson, 2017; Watkins, 2024; Reuters, 2024; O'Leary, 2020). However, not all cases are reported in the media, and privacy in cases of dismissal via ABS evidence limits access to court records. Clinician-researcher Dr. Barbara Cordell has reported at least four court cases in which she was directly involved as an expert witness, with ABS-related outcomes that included dismissal, reduction of charges, and the restoration of professional licensure (Logan et al., 2026). So far, outcomes have been driven by magistrates or prosecutorial decisions, so public details concerning individual parties and their medical records are limited. To the best of our knowledge, there has yet to be a jury acquittal in a DUI (or any criminal) case involving ABS. In any case, these rulings delineate a clear evidentiary pathway: validated assays, chain of custody, and controlled sugar/meal challenges make microbiome-mediated impairment legally cognisable.

In a defined subset, post-meal endogenous ethanol reaches levels that affect cognition, disinhibition, and mood, often time-locked to carbohydrate intake; ABS has been associated with irritability, hostility, and violence (Logan et al., 2024a). Contributors include recent surgery, antibiotics, ultra-processed sugar-dense diets, prescription and overt-the-counter acid-suppressants, and other ecosystem disruptors; post-viral dysbiosis (e.g., after COVID-19) may elevate risk in some profiles (Cordell, 2025; Yates and Saito, 2024). While early reports emphasized yeasts (e.g., *Candida* spp.), newer work shows that bacteria—notably *Klebsiella pneumoniae*—also generate substantial ethanol, with many ethanol-producing microbes expanding after antibiotics and high-sugar diets (Xue et al., 2023; Mbaye et al., 2024; Prescott and Logan, 2025).

It is important to emphasize that ABS, even if it is more common than currently appreciated, is a high-specificity pathway with a relatively simple linear and time-locked relationship between metabolite production and impairment. Courts are already well-equipped to understand ethanol as a chemical with cognitive-behavioral consequences, so translation is relatively easy. Such is not the case with other metabolites; ABS in the legal frame does not immediately equate to a generalizable template for microbiome signatures in culpability. While it may be the case that ethanol is only one among many microbially derived metabolites with cognitive–behavioral relevance, it may take years or decades before other individual metabolites can be proven to play a causal role in justice related behavior. Yet, as described below, the future of this area may involve metabolomic and multi-omics clusters than can reliably be linked to cognition and behavior. The broader point: ABS is likely a harbinger of further court–microbiome intersections.

## 6 Microbial signatures and multi-omics integration

Aided by large datasets and machine learning, researchers are beginning to identify gut-microbial signatures with diagnostic/prognostic potential. Emerging work suggests signatures can help identify autism spectrum disorders (Peralta-Marzal et al., 2024), ADHD (Richarte et al., 2021), and schizophrenia or bipolar disorder (Ioannou et al., 2024). In a recent study involving 68 adults with schizophrenia and 61 heathy controls, researchers reported that combined metagenome and metabolome analysis (based on 61 microbial species) allows for a highly accurate disease classifier, with an area under the curve of 0.94 (Huang et al., 2026).

These advances also involve breath testing. For example, preliminary work links metabolomic breath samples to cognitive function and behavioral disorders (Meurs et al., 2025; Jiao et al., 2023). With increasing sophistication, researchers are using breath testing as a way to link gut microbe derived metabolites to human diseases (Hernandez-Leyva et al., 2026). While still at an early stage, emergent research demonstrates that breath testing (capturing gut microbe produced volatile chemicals) has the potential to differentiate between schizophrenia, depression, and otherwise healthy controls (Henning et al., 2023).

Beyond disorders *per se*, nascent findings link microbial signatures to personality and temperament (Orme et al., 2025; Ueda et al., 2024; Sumich et al., 2022), impulsivity and risk-taking (Konstanti et al., 2024; Liu et al., 2024; Musculus et al., 2025), aggression-related traits including violent tendencies, self-harm, and reactive aggression (Chen et al., 2021; Deng et al., 2022; Costanza et al., 2025; Jakobi et al., 2024), and emotional capacities—regulation, stress resiliency, compassion (Ke et al., 2023; Faulkner et al., 2024; An et al., 2024; Nguyen et al., 2021). Early preprint evidence even suggests transferability of softer traits such as exuberant behavior between donor and recipient (Aatsinki et al., 2025). For now, this entire body of research remains at a very early stage. The available research is based on relatively small sample sizes and lacks cross-cultural diversity. However, when taken as a whole, it supports the robust preclinical mechanistic and fecal transplant findings. Experts in the field emphasize that narrowing the gulf between preclinical findings and diagnostic microbial signatures will require much more investment in multi-omics approaches, machine learning, methodological standardization, and inclusivity of diverse populations (Tegegne and Savidge, 2025).

For forensic relevance, omics-based technologies strengthen these signals by layering biomolecular readouts from blood, saliva, feces, or exhaled breath. Rather than relying on microbial features or single genes [e.g., the so-called ‘Warrior gene' (Farahany and Robinson, 2021)], polygenic risk scores can be combined with microbial signatures, measurable metabolites, and assessments such as neuroimaging (Hagenbeek et al., 2023). This will yield more robust composite risk contexts and allow for the integration of environmental considerations that shape treatment and rehabilitation. Multi-omics likewise clarifies how environments support mental wellness and how gene expression resists simplistic genetic determinism (Odintsova et al., 2023; Bohare, 2024).

Earlier hopes that neuroimaging alone would revolutionize legal reasoning were overstated: outside obvious pathology (tumor, gross structural damage), scans have had limited standalone explanatory power and remain peripheral in most criminal rulings (Greene and Cohen, 2004; Callender, 2021; Logan and Prescott, 2025). Patterns such as frontal underactivity with heightened limbic activity appear in some offenders—but also in many law-abiding individuals (Sapolsky, 2004).

We suggest that legalomics differs. The legalome promises explanatory power in select cases by integrating polygenic indices with multi-omics (genomic, epigenomic, transcriptomic, metabolomic), nutritional neuroscience, and microbial signatures (Logan et al., 2025c). Continued advances will help tease causal links between environmental factors (e.g., diet), behavioral outcomes (impulsivity, sensation-seeking), and microbial signatures (Konstanti et al., 2025), enabling a personalisation synergy not previously possible (Molla and Bitew, 2024). For the first time, expert teams can assemble multiple objective layers to provide biological context that goes well beyond an isolated neuroimaging scan (Tang et al., 2025; Hassan et al., 2025).

## 7 Challenges and ethical considerations

Legalomics sits at the intersection of responsibility, privacy, and bodily autonomy. Its value is not to biologise blame but to calibrate judgements with credible biology under clear guardrails. Biology is a bounded contributor, integrated with psychosocial context. To avoid fatalistic determinism, claims should stay mechanism-consistent and context-specific, emphasizing *sufficiency in receptive hosts* rather than global explanations. This stance strengthens the central thesis: law becomes more accurate and humane when it recognizes the individualized ways that biology—including neuromicrobiology—matters.

A preliminary distinction is essential. As noted in earlier sections, microbiome-related evidence divides into two categories of differing evidentiary maturity: *(a) bounded, testable claims* involving a single identifiable metabolite and a time-locked impairment pathway (exemplified by auto-brewery syndrome), and *(b) probabilistic, context-dependent markers* drawn from multi-omics signatures that correlate with behavioral phenotypes but remain vulnerable to confounding and population stratification. Category (a) can already meet courtroom standards of admissibility; category (b) cannot yet do so reliably, and responsible deployment of legalomics requires that courts, clinicians, and experts maintain this distinction rigorously.

### 7.1 Neurorights as governance

Microbiome, metabolome, and epigenome profiles are behavior-adjacent and sensitive. Ienca and Andorno (2017) identified four foundational neurorights—cognitive liberty, mental privacy, mental integrity, and psychological continuity—as the normative architecture for protecting individuals against unconsented access to, and manipulation of, neurally and biologically derived data. This framework extends naturally to legalomics: microbiome and multi-omics profiles can reveal health predispositions, dietary patterns, substance use, geographic history, and even intimate contacts, giving them a re-identification potential that parallels genomic data. Ligthart et al. (2023), in a multidisciplinary mapping of neurorights foundations, further specified that mental privacy protections must encompass any data from which mental states, behavioral tendencies, or cognitive capacities can reasonably be inferred. This is a criterion that behavior-adjacent microbiome profiles plainly satisfy.

To move from principle to practice, the following operational parameters should apply to any legalomics data collected in forensic, judicial, or correctional contexts:

### 7.2 Collection minimization

Collect only the analytes specified by a preregistered clinical or forensic question; broad-panel “fishing” is unjustified at the current evidence base.

### 7.3 Default retention

Data should be retained only for the duration of the legal proceeding plus a defined appeal window (we suggest a default ceiling of five years from final disposition, subject to jurisdictional variation), after which identifiable records are destroyed or irreversibly de-identified. This mirrors the data-minimization and storage-limitation principles of the EU General Data Protection Regulation (GDPR, Articles 5(1)(c) and 5(1)(e)) and analogous provisions in other jurisdictions.

### 7.4 Audit trails

Every access event—who accessed which records, when, and for what purpose—must be logged in a tamper-evident system. Access should be restricted to need-to-know personnel (treating clinician, instructed expert, designated court officer) with role-based controls and periodic compliance review, consistent with best practice in biobank governance (Staunton et al., 2022).

### 7.5 Secondary use prohibition

Data collected for a specific forensic or clinical question must not be repurposed for employment screening, insurance underwriting, unrelated criminal investigations, or population-level risk stratification without explicit, separate, and informed consent. Re-identification risks are non-trivial: Research demonstrates that gut microbiome metagenomic codes can uniquely identify individuals among populations of hundreds, with over 80% of subjects re-identifiable up to a year later (Franzosa et al., 2015).

### 7.6 Cognitive liberty and proportionality

Any biologically targeted sentencing condition—whether probiotic supplementation, dietary modification, or microbiome monitoring—must pass necessity and proportionality tests and remain voluntary and revocable under independent medical oversight. This constraint is especially important in custodial settings, where the inherent coercive pressures of incarceration compromise the conditions for genuinely autonomous consent (Ahalt et al., 2018). The 20^th^ century exploitation of prisoners in biomedical research is well documented. Accordingly, any legalomics-related intervention in a carceral context should require independent ethics review, a prisoner advocate separate from the legal process, a clear right of withdrawal without penalty, and ongoing monitoring by an oversight body that includes both clinical and legal representation.

### 7.7 Equity

Microbiome profiles track social determinants—nutrition access, sanitation, housing, marginalization, chronic stress—so policy should prioritize universal, non-stigmatizing improvements in custodial settings and avoid labels that compound discrimination. The justice system's eugenic past cautions against attaching biological “risk” to sociopolitical constructs like race (Arford and Madfis, 2022). Yet biology should not be elided because of that history (Elzein, 2024). Jurors and other decision-makers can punish more harshly when they perceive a biological condition (Berryessa, 2020; Allen et al., 2019), a pattern that may reflect science-literacy gaps (Thomaidou and Berryessa, 2022). The remedy is education, not a return to prescientific folk psychology. Any equity-monitoring protocol should track, at minimum, whether legalomics evidence is introduced differentially by race, socioeconomic status, or offense category, and whether its admission correlates with sentencing disparities.

### 7.8 Reliability and scope

Prespecified protocols, validated assays in accredited labs, chain of custody, known error rates, and observed replication are enablers—not barriers. Microbiome science, however, confronts well-documented reproducibility challenges that any courtroom application must address transparently. Variability in DNA extraction methods, primer selection, sequencing platforms, and bioinformatic pipelines can generate divergent taxonomic profiles across different laboratories (Sinha et al., 2017; Kool et al., 2023). Batch effects—systematic technical variations unrelated to biological signal—are pervasive in omics research and require purpose-built correction methods; tools developed for gene-expression data may not adequately address the compositional and zero-inflated structure of metagenomic count data (Yu T. et al., 2024). The STORMS (Strengthening the Organizing and Reporting of Microbiome Studies) checklist, published in *Nature Medicine* as a consensus reporting standard, provides a minimum transparency framework that legalomics submissions should adopt (Mirzayi and Renson, 2021).

### 7.9 Practice integration

Add a brief GI/antibiotic/sleep screener when symptoms flag; use targeted referral rather than broad panels; phrase conclusions as bounded, evidence-tethered contributors; and upgrade institutional basics—sleep windows, nutrition, infection control—where they improve stability and wellbeing at scale. Framed this way, legalomics functions as a disciplined, rights-respecting upgrade to fairness, deployed only where evidence and ethics align.

### 7.10 A note on terminology

The terms “competency” and “competence” carry distinct connotations across jurisdictions. In United States criminal law, “competency” typically refers to a defendant's present capacity to understand proceedings and assist counsel (the Dusky standard; *Dusky v. United States*, 362 U.S. 402, 1960), whereas in English and Australian law, “fitness to plead” or “fitness to stand trial” serves a functionally analogous role under different doctrinal language. In forensic and legal psychology more broadly, “competence” is used as the generic capacity term (Melton et al., 2017). Throughout this article, we use the terms interchangeably unless specifying a particular jurisdiction's doctrinal framework; readers should note that the underlying construct—the ability to participate meaningfully in legal proceedings—is substantively consistent across common-law systems, even where the procedural thresholds differ.

## 8 Future directions

To move from promising thesis to serviceable practice, legalomics must feel familiar and useful to clinicians and courts. The goal is not to replace psychosocial formulations but to calibrate them with credible gut–brain biology where it genuinely changes decisions. Within the ethical guardrails above, we prioritize the following (Box 1).

Box 1 **Legalomics implementation checklist.**

The following checklist consolidates operational safeguards across five domains. Each item specifies a responsible actor and an indicative standard.

**Table:** 

**Item**	**Safeguard**
**I. Clinical screening and referral**	
**1**	Symptom-triggered screening. When a forensic or legal-psychology evaluation identifies persistent GI symptoms, recent broad-spectrum antibiotic use, unexplained cognitive–behavioral fluctuations, “brain fog” or carbohydrate-linked mood/impairment episodes, the evaluating clinician should add a brief GI/antibiotic/sleep screener and, where warranted, refer to a gastroenterologist or microbiome-informed specialist. Screening should be targeted, not routine.
**2**	Role clarity. The forensic psychologist/psychiatrist identifies clinical flags and frames the legal question; the referring clinician or specialist conducts or orders biological testing; and the laboratory performs and reports the assay. No single actor should span all three roles.
**II. Laboratory testing standards**	
**3**	Accredited laboratory, validated assay. All microbiome or multi-omics analyses submitted as evidence must be performed in a laboratory holding relevant accreditation (e.g., CLIA, ISO 15189) using assays with documented sensitivity, specificity, and known error rates.
**4**	Standardized protocols. Sample collection, storage (temperature, preservative), DNA extraction, sequencing platform, and bioinformatic pipeline must follow a prespecified protocol and be fully disclosed. Conformity with STORMS reporting standards (Mirzayi and Renson, 2021) is recommended as a minimum transparency threshold.
**5**	Chain of custody. Biological samples must be handled under forensic chain-of-custody procedures from collection through analysis, with tamper-evident seals, time-stamped transfer logs, and named custodians at each stage.
**III. Courtroom communication and limits**	
**6**	Bounded conclusions. Expert testimony must distinguish between bounded, testable metabolite–impairment pathways (e.g., endogenous ethanol in ABS) and probabilistic multi-omics associations. In most cases conclusions should be phrased as evidence-tethered contributions to a biopsychosocial formulation—not as standalone determinants of culpability or competence.
**7**	Admissibility thresholds. In jurisdictions applying Daubert (US), the expert should be prepared to address testability, peer review, error rates, standards, and general acceptance. Equivalent reliability frameworks (e.g., Criminal Practice Directions in England/Wales, Mohan/Abbey criteria in Canada) apply elsewhere.
**IV. Governance safeguards**	
**8**	Data minimization, retention, and audit. Collect only prespecified analytes; retain identifiable data for no longer than the proceeding plus appeal window (default ceiling: 5 years); log every access event; destroy or irreversibly de-identify records upon expiry. Secondary use for employment, insurance, or unrelated investigations requires separate informed consent.
**9**	Custodial consent protections. In carceral settings, any legalomics-related data collection or intervention requires independent ethics review, a prisoner advocate independent of the legal process, a clear and unpenalised right of withdrawal, and ongoing oversight by a body with both clinical and legal representation.
**V. Equity and fairness monitoring**	
**10**	Disparity tracking. Jurisdictions or institutions adopting legalomics evidence should monitor whether such evidence is introduced differentially by race, socioeconomic status, or offense category, and whether its admission correlates with sentencing or dispositional disparities. Results should be reported periodically to an independent oversight body.

### 8.1 Causal mapping that matters to practice

Link specific biological pathways to clinician-recognized constructs—impulsivity/delay discounting, affective volatility/stress reactivity, inhibitory control/decision thresholds—by pairing microbiota transfer or other perturbations (antibiotics, diet, sleep) with forensic-resonant behavioral read-outs, while tracking intermediates (HPA-axis tone, microglial activation, SCFAs, tryptophan–kynurenine metabolism). The exposome (i.e., accumulated experiences and exposures over time) must sit alongside biology (infection, medication, nutrition, pollution, greenspace, sleep, trauma, social connection). Deliverable: a translational chain from microbe/metabolite → intermediate brain process → legally relevant behavior under specified conditions.

### 8.2 Outcomes courts already understand

ABS shows how a known molecule (ethanol) shortens the path from gut to behavior. Future legalomics should be preregistered, adequately powered, and test whether adjunctive interventions (nutrition, sleep regularity, infection control, psychobiotics under medical oversight) reduce decision volatility, moderate affective lability, improve adherence, or stabilize behavior in custody. Endpoints should include risk-taking/inhibition tasks, clinician-rated volatility, disciplinary incidents, and post-release engagement; chain-of-custody and assay quality systems are essential for Daubert/Frye scrutiny.

### 8.3 Workforce education

Forensic psychologists will need training that integrates lifestyle factors tightly coupled to the microbiome. The American Psychological Association has urged members to stay current on links between diet and mental health risk (DeAngelis, 2023). The American Psychiatric Association has recently emphasized nutrition in mental health promotion and care (Merlo et al., 2025). Because diet shapes the microbiome—and diet–mental-health links are at least partly microbiome-mediated—basic literacy is necessary (Horn et al., 2022).

### 8.4 Minimal, proportionate screening

Scope-respecting toolkits should flag when referral is warranted. For ABS, brief questions on recent antibiotics, persistent GI symptoms, carbohydrate-linked mood swings, major diet shifts, sleep disruption, glucose dysregulation, and acid-suppressant use can trigger a standard pathway: supervised carbohydrate challenge; serial venous alcohol by headspace GC (paired breath if required); glucose tracking; exclusion of exogenous sources; repeatability.

### 8.5 Decision support beyond heuristics

Layer low-burden biological indicators (sleep regularity, simple inflammatory indices, metabolic stress) onto psychosocial variables to assist opinions on competency, mitigation, and supervision. Tools must be assistive, calibrated, fair across groups, and transparently monitored for drift and bias.

### 8.6 Systems-level, low-regret trials

Correctional health can immediately improve nutrition quality, reliable sleep windows, infection control, and stress-reduction routines via stepped-wedge/cluster designs—without invasive testing. Metabolomics/microbiome data can verify whether systems provide adequate nourishment (Ma M. et al., 2025) or predominantly ultra-processed “food” (Abar et al., 2025). Institutions already track outcomes (violent incidents, medical visits, disciplinary actions, staff wellness); positive results justify scaling-up and eventually, if evidence permits, policy changes. Common infrastructure—cross-jurisdiction registries with harmonized metadata, open pipelines, plain-language bench materials for courts, and interdisciplinary training—will determine integration and usefulness.

### 8.7 Beyond forensics: legal psychology

Legal psychology spans pre-screening of law-enforcement candidates, wellness across the courtroom workgroup, applied prevention in justice organizations, and rights-based carceral conditions. Burnout among legal and law-enforcement professionals reflects accumulated stress and lifestyle factors (Queirós et al., 2013; Krill et al., 2023). Nutrition and the microbiome are emerging upstream levers for risk and performance (Penttinen et al., 2021; Kipfer et al., 2025); legalomics can guide universal and personalized support programmes (Logan et al., 2025d). Objective biological markers may minimize the shortcomings of ‘paper-and-pencil' self-reports and subjective clinical impressions as currently used in preemployment screening and fitness-for-duty assessments (Oprinca-Muja et al., 2026).

### 8.8 Carceral environments as “dysbiotic by default”

Excessive noise, artificial light at night, ultra-processed foods, and social isolation are linked to gut-ecosystem disturbance; emerging evidence ties these conditions to neuropsychiatric symptomatology via the microbiome (Yi et al., 2025; Feng et al., 2025; Yang et al., 2025; Yuan et al., 2025). Carceral populations show distinct microbiota profiles (Duan et al., 2022; Langmajerová et al., 2025). Future work should disentangle which environmental components drive biological harms/benefits and how to remediate them. For example, multiple studies suggest that healthy dietary patterns are associated with cognition and behavior that would minimize the risk of justice involvement and/or limit aggression or antisocial behavior in carceral settings (Gibbs and Beaver, 2026; Prescott et al., 2024). While select nutrients can have direct and indirect influences on brain structure and function (e.g., omega-3 fatty acids) (Raine and Brodrick, 2024), others may be operating through the gut microbiome (Ferri et al., 2026).

### 8.9 Reexamine existing research

Advances in legalome science will allow researchers to scrutinize well documented findings through a new lens. For example, multiple studies have linked early pubertal timing to higher risks of delinquency and subsequent adult criminality. But what causes early pubertal timing? Emergent research indicates that exposure to environmental chemicals causes gut dysbiosis, which in turn leads to early pubertal timing; in preclinical work, fecal microbiota transplantation from early puberty donors (previously exposed to low-dose endocrine-disrupting chemicals) into germ-free mice recapitulated early pubertal onset, supporting a causal role for gut microbiota (Wu et al., 2025). Another example includes pharmaceutical risk profiles. For example, the restless leg syndrome drug ropinirole and other dopamine agonists have been consistently linked to risk taking and addictive behaviors (Wolfschlag and Håkansson, 2021). The intersection of ropinirole prescriptions and justice involvement has been described in recent reporting by the BBC (Titheradge, 2026). These ‘side-effects' do not occur in all users, and the onset of gambling and hypersexuality, for example, often trails drug initiation by more than a month (Obata et al., 2025; Cornelius et al., 2010). Why? Recent research indicates that this drug class may cause significant disturbances to the gut microbiota (van Kessel et al., 2022).

### 8.10 Forensic neuroecology and the anthropocene

In recent years, multiple studies have linked climate change and environmental degradation-related factors with increased risks of violence and criminality. For example, airborne particulate matter and heat events are linked to interpersonal violence and crime (Awaworyi Churchill et al., 2023; Yao et al., 2025; Berman et al., 2019; Choi et al., 2024). Exposure to heavy metals such as lead and cadmium have been linked to aggression, antisocial activity, and criminality (Shaffer et al., 2025; Logan et al., 2024b). The mechanisms by which these environmental factors influence cognition and behavior are poorly elucidated. However, emergent research shows that airborne particulate matter (Filardo et al., 2022), heat events (Huus and Ley, 2021), and heavy metal exposures (Arun et al., 2021), have the potential to alter the composition and diversity of gut microbiota. Enhanced understanding of potential mechanistic pathways may help identify vulnerable and at-risk populations.

## 9 Conclusion

Forensic and legal psychology already work from a biopsychosocial frame; gut–brain science calibrates (not replaces) that tradition. Under defined conditions, microbial states can nudge behavior-relevant brain processes; in rare but dispositive instances they can produce legally cognisable impairment (as in well-documented auto-brewery cases). Hence the case for legalomics: disciplined, neurorights-aware use of omics as a bounded contributor to truth-seeking—neither alibi nor oracle.

Translation demands practical supports: clinician education, proportionate screening and clear referral pathways; courtroom standards that render microbiome claims testable and fair; and correctional “low-regret” upgrades—sleep, nutrition, acoustics, lighting, infection control—with collateral health benefits. Progress also requires investment in studies that trace microbe → mechanism → behavior, use outcomes judges recognize, and are underwritten by shared infrastructures so laboratories, clinics, and courts speak a common language.

If the law's calling is to align responsibility with reality, then disciplined attention to neuroecology may, if the research continues to advance, be a duty of care. Microbiome and behavior evidence is already entering courtrooms via ABS; the task now is stewardship, thoughtful research designs, and advance planning for ethical frameworks that may be required. Realized within a neurorights frame, legalomics can make justice more accurate and humane—and better oriented to genuine restoration.

## Data Availability

The original contributions presented in the study are included in the article/supplementary material, further inquiries can be directed to the corresponding author.
